# Book Reviews

**Published:** 1817-03

**Authors:** 


### CRITICAL ANALYSIS
### *OF RECENT PUBLICATIONS*
### IN THE
### DIFFERENT BRANCHES OF PHYSIC, SURGERY, AND MEDICAL PHILOSOPHY.

*On the Diagnosis, Nature, and Treatment, of the Hooping Cough;* by ADALBERT FREDERICK MARCUS. Bamberg and Leipzig, by Kanz, 1816, XVIII. and 216 pp. 8vo. Extracted and translated by Dr. VON EMBDEN.

THE well-known and learned author, after labouring for many months under a disorder equally painful and dangerous, thinking himself in the way of recovery, on the 23d of April, returned thanks to Providence and his physicians in the Preface to the present publication, which, on account of his debility, he was obliged to dictate; and, on the 25th, this great philanthropist and practitioner, who, for the last twenty years, acted so conspicuous a part in the reformation of medicine, terminated a career equally active and useful on the very day when the present work was to have left the press, leaving his biography to an abler pen. We select only the most essential passages from the present work, in order to put our readers in possession of the leading features of a publication in which it was the late author's wish to fix the identity of bronchitis and hooping cough.

The name of a disorder should, as far as possible, describe the nature and seat of the same. Dr. M. calls hooping cough

*bronchitis*

*bronchitis epidemica*, confident that its seat is in these parts, and its nature inflammatory. The hooping cough is not to be considered as a new disorder; but must be counted among those, in which neither the seat, the affected organ, nor the influences, are of such a nature that it should not have prevailed at all times. It belongs to those disorders, which, in some parts of the world, appear very seldom, and which, with regard to their periodical return, spare many parts for a long time. Intending to show the identity of bronchitis and hooping cough, the author gives a picture of both. [It would have been more to our wishes, if, after describing the two, he had pointed out the difference, which, in our opinion, would not have been difficult.—Edit.]

Hooping cough has been described much more frequently and exactly than bronchitis. Many symptoms seem peculiar to the former, from their occurrence in childhood, and because many phenomena depend on the tenderness and softness of the infantile organism. Bronchitis has the same run with the hooping; though sometimes it is quicker, at others slower. Both of them wear the stamp of a catarrh, with all the concomitant symptoms; in neither of them does the stitching pain in the side exist, or, at least, it is but temporary; in both of them the cough follows a deep inspiration; the difficulty of respiration is instantly aggravated in both when lying down; the coughing paroxysms are the same in both, the exacerbations periodical, and the patients apparently well in the intervals. The expectoration is similar; in neither of them is hoarseness of any consequence or duration; hooping is a chief symptom in bronchitis, and it gives the name to the other. The sudden transition from inflammatory activity to incurable debility is common to both. The dreadful struggle in the last hours of life is the same in both morbid forms; and many other symptoms, though not essential, are common to both.

Dr. Watt, of Glasgow, in his Treatise on the History, Nature, and Treatment, of Kink Cough, 1812, first hinted at the identity of the two disorders. Having lost three of his own children in the hooping cough, dissections and a comparative view of the symptoms of both disorders, in a manner convinced him that the seat of both was in the bronchiæ. Before his time, the disease was considered as a purely nervous and spasmodic state. Few ventured to conjecture what might induce this spasm: miasma and contagion cannot be considered as proximate causes, as they do not constitute the disorder itself. If hooping cough be considered identical with bronchitis, its nature must be inflammatory.

But

But, Dr. Albers having disputed this doctrine of Watt, it is necessary we should give here his objections, together with Dr. Marcus's refutation.

Dr. Watt maintains, bronchitis does sometimes arise suddenly, but sometimes hides itself, and only appears gradually. Dr. Albers says, "hooping cough never appeared so suddenly, nor yet does it remain so hidden as bronchitis;" to which Dr. Marcus replies, "that a distinction ought to be made between a sporadical and an epidemical disorder,—bronchitis being of the first kind, but hooping cough of the latter; the development of both must be sometimes quicker and sometimes slower. Though the sense of weight, combined with quick and oppressed respiration, were symptoms of bronchitis alone, Dr. A. could, nevertheless, not consider this as a proof for his argument; as here, likewise, the difference above-mentioned takes place, Dr. A. asserts, hooping cough to be a disorder of the pectoral nerves, and not essentially combined with inflammation. But, if hooping cough, as Dr. M. intends to prove, is a catarrh, it unquestionably must be combined with it. If it be farther true, that fever be founded on inflammation, hooping cough must of course be inflammatory, as it never does exist without fever. Dr. A. in maintaining that hooping cough belongs to the class of epidemical disorders, tacitly admits its being of the febrile inflammatory kind. It cannot serve as any proof against the aoove assertion, that children sometimes recover from hooping cough without medical assistance, for, in every disorder, there is also a tendency of nature towards recovery. Notwithstanding Dr. A. is of that opinion, Dr. M. thinks this no impediment to considering bronchitis and croup catarrhous disorders. The catarrh is seated in the trachea and the bronchial system; and, even if we suppose some of these disorders to be seated in the vascular system, and some in the mucous membrane and its glands, the inflammatory nature of the hooping cough is by no means refuted by it; for the mucous membrane consists, for the greatest part, of the rete vasculosum, and can thus not suffer under inflammation without the blood vessels participating in it, and *vice versâ*.

Though the seat and nature of a disorder is already sufficient for fixing its character, Dr. M. nevertheless adds a few more particulars on the character of the hooping cough. The first point constitutes its catarrhous quality. It is accompanied with a cough from its beginning to the end, which always forms its character, particularly if combined with expectoration. Some authors, however, assert the catarrh to be only a forerunner of the hooping cough. But there is not a single symptom which unfolds itself in the hooping hooping cough, which, in some degree, has not already existed in the catarrhous stage, except that in a later stage each is better marked. Almost all inflammatory pectoral affections have a slow progress in the beginning, but become formidable in the acme of the disorder, though the lymphatic inflammations mostly run a shorter race than the rest.

It may appear astonishing, that an inflammatory state should be of such a long duration; however, there are pulmonary consumptions, which, likewise, are very slow in their progress, though they never abandon their inflammatory character. Neither is it necessary, that the hooping cough should continue inflammatory the whole time of its course. Dr. M. now, with much probability, from the seat, nature, and character, of the disorder, concludes upon its identity with bronchitis, which point is still more confirmed by dissections.

Earlier writers, who dissected subjects that died of the hooping cough, do not mention inflammation or any other particular change in the bronchial system; they found an enormous collection of air in the lungs, a great deal of water in the pericardium, the diaphragm strong, firm, and folding, the muscular part much distended with blood, the phrenic artery plainly visible, and the veins turgid with blood. Dr. Watt's conclusion, from the examination of his own children, is confirmed by two dissections performed by Dr. M. In one of them, he found, in the place where the trachea divides itself, slight inflammation and traces of puriform matter. On the further progress, the ramifications of the bronchiæ appeared more and more inflamed, gangrenous, and of a brownish red colour, the vessels formed livid webs, filled with puriform matter to such a degree that it was a matter of surprise how air could at all penetrate them. In the other case, water was found in the cavity of the breast; the larynx and trachea contained a fluid puriform phlegm; the lower part of the trachea was of a slight red tinge; the deeper the tracheal ramifications descended, their colour changed more and more to a dark red, and they were entirely filled with a frothy pus like matter, which issued from them in great abundance, and seemed to fill them up quite to the air-cells.

The result of both dissections is, that the hooping cough originates in bronchitis, or, in other words, that the hooping cough is the consequence of this inflammatory state.

It might, perhaps, be objected, that a few dissections cannot yield any decisive proof upon such an intricate subject; however, if the topical suffering, the phenomena, progress, issue, and treatment, agree with the pathological dissections; tions; few examinations may suffice, to give the greatest degree of probability or certainty to such a fact. On comparing the results of the dissections of such subjects as died of the bronchitis, several of which are to be found in Badham's Treatise upon this subject, the assertion, that bronchitis and the hooping cough are identical states may acquire additional confirmation. It cannot be contradicted, at least, that the seat and form of both complaints are in perfect unison.

The hooping cough, when completely formed, is easily known, but exceedingly difficult to be discovered in its first beginning. When it appears as a convulsive cough, it has reached its acme. The pathologico-semiotical object is, to distinguish it in its first development. If once a complaint has attained its highest pitch, art can do but little against it.

The peculiar symptoms of the hooping cough are, its peculiar sound, the coughing fits succeeding each other with great rapidity; the quick expirations that leave the patient no time for breathing, and threatens to suffocate him; the continuance of such an attack till retching and vomiting a plegm-like matter takes place. We shall offer a few words towards the elucidation of these symptoms.

The bronchial contains the greatest abundance of vessels, in which arterial exceeds the venous system, though it is also sufficiently provided with veins, lymphatics, and nerves. Such a texture has a tendency towards inflammation, which may assume a very great variety of characters. In every inflammation, fever is the most essential symptom; this is also the case in the hooping cough. The mucous membrane, where this disorder has its seat, belonging to the lymphatic system, and containing a considerable texture of lymphatic vessels, besides a great number of glands, it results from it, that the fever attending the hooping cough commonly bears an intermittent type; but the vascular web being primarily affected in this disorder, the fever can also amount to synocha. The hooping cough is undoubtedly a febrile state, as no catarrh exists without fever, and cases where the attending fever is but imperceptible, are to be considered only as exceptions. The free intervals in the hooping cough have led to a deception; but, if it cannot be denied, that inflammation is the foundation of intermittents, it is plain, that inflammation and fever may also be present in the hooping cough. From its having its seat in a lymphatic texture, results also the long duration of the complaint and the fever attending, as also the tendency of this fever, to make its reappearance in case of a relapse, as synocha.

The whistling tone, as also the cough in general, are pathognomical thognomical in the hooping cough. Considering the cough to be an appearance accompanying the disorder from the beginning to the end, the hooping cough must be pronounced a pure catarrhous affection. Only where the trachea partakes in the pulmonary sufferings, the cough appears; consequently, the seat of the complaint ought to have been looked for no-where else, but in the trachea itself. As soon as the air cannot pass through the ramifications of the bronchiæ, a discordance in the tone must arise. In what manner are then the expirations that follow each other with such rapidity to be accounted for? The cough does not take place till the passage of the air through the bronchiæ is obstructed, which obstruction is caused by the matter morbidly secreted and accumulated in the bronchiæ, to such a degree, that being forced into the trachea it continues to irritate till expelled by struggling, coughing, or vomiting. The rapidly succeeding expirations result from the first impulse for clearing the bronchiæ of the matter accumulated in them; being once given, this impulse admits of no cessation till the expulsion of whatever is there accumulated succeeds; in case that the power exerted is not sufficient for this purpose, the attack returns again directly. The rapidly succeeding expirations preventing the inspiration, give rise to that singular tone of the cough from which its name has arisen.

Dr. M. next proceeds to the consideration of the different stages of the hooping cough. Three are generally admitted, —the catarrhous, convulsive, and the declining stage of the disease. But, according to the doctor's opinion, the convulsive stage does not materially differ from the catarrhous, the former only exhibiting the complaint as forming, and the latter in its completely formed state. For even in the stage of formation, some indications of the second period appear. On observing the cough more closely, it already bears the stamp of what is called the hooping cough, viz. it takes place at considerable intervals, and is of long duration. The second stage takes place on the seventh or fourteenth day at farthest, and is only to be considered as an increased inflammatory condition of the bronchiæ. The appellation of stadium convulsivum is unfit, erroneous, and in some respects, even dangerous. According to the law of the succession of the appearances, no other symptoms can occur in the third stage than those that announce the commencement of it; these are confined to the catarrhous affection and the cough, and no traces of fever ought any more to exist.

The hooping cough being an epidemical complaint, it must be caused by the influence of the climate, and, of course, by any inflammatory febrile condition. A sporadical hooping cough does but rarely occur, and then it deserves more the appellation of bronchitis.

Which are the predisponent momenta producing the hooping cough?—The prevalent wet cold state of the weather seems to lay the foundation for a predisposition towards this complaint, therefore it developes itself particularly in spring and autumn. The infantine age may also with justice be classed amongst the predisponent causes.

Is the hooping cough to be considered as contagious?—Dr. M. is of opinion, that no epidemical disorder exists which, in course of time, may not assume a contagious character. But the contagion does not occur before the beginning period of morbid excretion; this period taking place sooner or later in different disorders, the contagion follows in the same proportion. The contagious matter seems to be nothing else than one of the gazes in the animal organism in an altered state. Dr. M. therefore, considers the hooping cough as a contagious disorder, but where no proper miasma is to be supposed. But, where the atmosphere is impregnated with the effluvia of a hooping-cough patient, infection probably takes place in such as have a predisposition towards it.

The hooping cough, by a fixed crisis, either passes over into health or into other disorders, and death. When properly treated and attended to in the first period, the crisis comes on with sweating, sediment in the urine, and expectoration. On account of the complaint being so deeply seated, the crisis in the most favourable cases does not take place before the fourteenth or twenty-first day. The secretion of phlegm in the tracheal system being less in the hooping cough, expectoration is wanting in the first stage, and the cough is dry. As soon as expectoration takes place, the inflammation and topical suffering are lessened; the crisis itself consists in the expectoration being brought to maturity. The secretions disturbed in the progress of the complaint are those of the skin, the renal system, and the intestinal canal. If the crisis take place in the first period, a favourable change in the urine is previously observed, the skin becomes moister, the cough decreases, and expectoration succeeds; thus the hooping cough, by increased attention and proper treatment, finishes critically with the fourteenth or twenty-first day. The opinion, that the hooping cough requires six, nine, and twelve, weeks for its termination is erroneous.

If no crisis take place during this period, and in the above manner, the symptoms encrease, and assume the character racter of what is called the suffocating stage. The suffering of the bronchial system attains a higher pitch; the inflammation increases and spreads farther; the vessels remain turgid with the stagnating humours; the crisis is uncertain and protracted. Whatever occurs in the later progress, belongs to the imperfect, or, at least, to the partial crisis.

A hooping cough not critically decided, passes over into other complaints, particularly the phthysis pituitosa, rhachitis, atrophy, scrofula, dropsy, &c. and sometimes death. There are divers specifics recommended against the hooping cough; but, if a more general and rational method of curing this disorder can be adopted, these specifics will soon be done away with.

Whoever considers the methods of cure, adopted at all times against this complaint, will be convinced, that the antiphlogistic process has never been entirely rejected by any observer. The physicians of former times, who more purely followed up the course of observation, almost without exception favoured this process, even to the most copious venæsections; and only the opinion of the nervous and spasmodic nature of the hooping cough, caused the introduction of what are called antispasmodics. The most convincing proof, that none of these praised remedies were actually specific, is their frequent change. But, the greatest disadvantage in the treatment rested in the opinion, that the catarrhous stage was considered as essentially different from the spasmodic. The epidemical and contagious condition of the disorder ought, long ago, to have recommended the antiphlogistic method. Had they been earlier convinced of the truth, that every epidemical contagious complaint ought to be treated in an antiphlogistical manner, thousands of victims would have been saved.

Dr. M. considers what has been called the spasmodic stage, as the increase of the inflammation, and, therefore, insists upon the application of the apparatus antiphlogisticus in its full extent. However, it still remains a difficult task to determine, when or where this remedy or another is to be applied, the succession in which they are to follow, and how the transition from the one to the other is to be made.

The vigorous antiphlogistical method which Sydenham and Huxham, from manifold experience, taught, consisted in blood-letting, gentle purges, and low diet; these were relinquished upon erroneous theoretical principles, by men who did not believe that a complaint of such a long duration could consist of inflammation; but, could the pathologist have supposed hooping cough to be identical with bronchitis, the antiphlogistical process would long ago have been re-introduced.

duced. Hooping cough and bronchitis being, in Dr. M.'s opinion, the same disease, a few words on the cure of the latter introduce the special treatment of the former. According to Badham, blood-letting is the first grand remedy in cases of bronchitis, when the powers are still unimpaired, the inflammatory symptoms clear; yet, it has not always the same wished-for effect, as in other acute pulmonary complaints. The reason of which is, that, in the latter, we have to cope with a diathesis phlogistica, and, in the former, with a diathesis catarrhalis. There is a material difference between the irritability of the arteries and that of the lymphatics; when the latter are too much deprived of their energy, they will, in the later progress of the complaint, be reduced to a state of debility.

The quantity of blood to be drawn is to be adapted to the circumstances of every particular case. Whether blood-letting is to be repeated, must be determined by the condition of the blood taken away, and the consequences attending the same. Badham recommends the continued application of antimonial remedies, and calomel given frequently and in small doses. Expectorants are not much praised, though they are not entirely rejected. The application of emetics, in the last asthenic stage of bronchitis, is said to be of decisive effect. Opium ought to be given with great caution, and nothing is said to be so beneficial as change of air. Whatever Badham and others are recommending, in what is called acute and asthenic bronchitis, is applicable in hooping cough, and also in the same order.

It depends upon the greater or lesser violence of the first period, how the antiphlogistic process is to be managed. As soon as the complaint is distinctly known, every occasional cause that may increase the catarrhous inflammatory condition must be removed. The successful treatment of the complaint depends upon the good and proper management of the catarrh. Equality of temperature is the first condition required; the children must be kept in the room till the crisis takes place, by expectoration and perspiration. In case the attending fever is of importance, they must be kept in bed. It is owing to the neglect of this care, that the hooping cough, beginning at the weaker degree of inflammation, rises to its highest pitch.

All kinds of nutritious irritating food and beverage must the more carefully be set aside, the better the appetite, and the stronger the patient's desire is for food. But, if in this period the fever is imminent, remedies must be applied, amongst which, blood-letting takes the first place. Sydenham and Huxham, as soon as they distinguished the complaint, instantly proceeded to repeated venæsections with the greatest success. Though the infantile age does not admit them in the same proportion as adults, nevertheless it cannot, even in children, be entirely dispensed with. The constitution of the body and the weather, indeed, decide, yet only with regard to the greater or smaller quantity. Venæsections, according to Dr. M., are a condition *sine qua non* in the hooping cough, if the same is to be shortened and brought to an easy and happy issue. The threatening form in which it appears at a later period, is a consequence of the neglected venesection, which is indicated as soon as the local suffering begins to be general. If the child has passed its third year, venæsection may be had recourse to without any hesitation. For children in their first year, it is rarely requisite; the effect may be attained by means of leeches, applied in sufficient quantity, and as near to the suffering part as possible. The internal remedies must be of the antiphlogistic class; to these belong the kali nitricum, sal ammon., tartarus depuratus, the neutral salts, the liquor acetite. Nitre is particularly useful when the fever approaches to the inflammatory character. Minderer's spirit, which in part supplies the place of sal. ammon., and which, with a decoction of marshmallow, is so applicable in children, are among the best remedies.

In almost all catarrhous affections, and, of course, also in the hooping cough, Dr. M. finds the mixtura oleosa exceedingly beneficial, but it must be fresh prepared every day. Expectorants in this stage are not proper.

Under this treatment, the abatement of the fever takes place towards the eleventh or fourteenth day, and, by the twenty-first, the disorder is at an end; the children, however, still remain for a long while in the disposition for it, and easily relapse.

In case the hooping-cough does not take this course, which, under an improper treatment, was hitherto but seldom the case, it appears in that form which has been described by the observers in the most glaring colours. The inflammatory condition has now attained a much higher degree; and suffocating symptoms now appear. These deceived the physicians, and misled them into erroneous opinions; but nothing has been more detrimental to the management of this complaint than the rage for finding out specifics against it. One must be convinced of the disorder not having as yet formed a transition, but that it is only striving to attain a higher degree of inflammation. When the error, that the complaint was only now beginning, prevailed, less efficacy was employed than the imperious moment required. In this this stage, Dr. M. finds blood-letting particularly indicated: if not yet applied, not a moment is to be lost in instituting it. When blood-letting is imperiously indicated, and has been neglected at an earlier period, it must now be applied copiously: insufficient disproportionate venæsection frequently gives the blood greater force to rush with violence towards the affected part.

The first venæsection must be free, and a pound of blood may, without hesitation, be taken away. If children above three years old should even faint away during the operation, one must not be deterred, but only keep the vein shut for a moment, and then draw off as much blood as was conceived necessary. The condition of the blood, and the abatement of the symptoms, must decide whether the operation is to be repeated. In pulmonary complaints, there is also a debilitas vitalis, originating in the interrupted functions of an organ so requisite for life; and therefore ought not to deter us from blood-letting. In the hooping-cough, the children frequently lie quite exhausted, with a pulse suppressed and small; but, as soon as the operation has taken place, all these appearances change. Even in the last stage, Dr. M. has seen venæsection applied with success.

Wherever indications for blood-letting occur, the application of the whole apparatus antiphlogisticus has also its place. These remedies are to be continued till the more violent degree of inflammation is removed. This may be known to be the case, 1st, by the decrease of the fever, and its transition from the continual to the intermittent type; and, 2dly, by the condition of the expectorated matter. If the latter is perfectly maturated and copious, the inflammation has already considerably decreased: the antiphlogistic method, however, ought not to be entirely set aside.

Dr. M. declares emetics to be superfluous and objectionable. Their application rests upon an erroneous supposition, and has experience against it. They are, however, conditionally applicable in hooping-cough,—1st, where the stomach suffers; 2dly, where suffocating fits occur, when the powers of nature are too weak to throw off the tough matter accumulated in the pulmonary cells.

Purges are more applicable, but they must be selected from the antiphlogistic class.

Mercury decidedly is to be ranked amongst the chief of the remedies indicated in the hooping-cough, partly as being an antiphlogistic, and partly as belonging to the class of resolvents. The question only is, at what period it is applicable? Venæsection must have preceded, and the fever be abated.

abated. The best form is either in the shape of a powder, or in a fluid state, as solutio mercurialis gummosa.

Antimonials are also not to be rejected. Mr. M. however, prefers the vinum antimonii to the precipitated antimony and mineral kermes. But these remedies are only to be applied when the inflammation is already considerably abated, and on the decrease, and when the object is to affect the secretory organs.

Much was expected from the use of musk; but it is useless, if not injurious.

Opium, so excellent a remedy on other occasions, is contra-indicated here; for, though it suppresses the cough, and makes the children enjoy a quiet night or two, yet it makes them stupified: they start in their slumbers, and symptoms of peripneumony soon appear.

Hyoscyamus operates in a similar manner; and the same may be said of all the other narcotics.

Though the olid gums are not so objectionable as the narcotics, yet they are not applicable at any period of such an inflammatory condition as the hooping-cough is. If the hepar sulphuris is actually of any service, it can only be at a later period of the complaint. Neither much increasing the inflammatory condition, nor suppressing the expectoration, it belongs not to those remedies which are directly contra-indicated.

The first and second stage of this complaint having been properly treated, it will rarely be the case that remedies are requisite in the stadium decrementi. The morbid secretion in the bronchiæ alone is still going on; a state of debility has taken place, as a consequence of every inflammatory state. Great caution, however, is necessary against the too precipitate use of corroborants. The disposition to inflammation is still existing, and easily re-kindled. At this period the Peruvian bark is indicated, but must not be applied in any other form than that of the decoctum aquosum. This period must be treated very diætetically.

Should this period, which commonly lasts four days, be protracted to too great a length, there is some fear lest the hooping-cough might pass over into a phthisis, which leaves not much room for favourable expectations. In this case, the cinchona, together with the moosa, digitalis, polygala, myrrh, hyoscyamus, opium, milk diet, and change of air, will render the best services.

---

We are favoured with the above account by our valued correspondent Dr. Von Embden. We shall take the liberty of concluding it with a summary of our own practice, confirmed firmed by the experience of thirty years, and by conversation with the London practitioners.

The first difference between hooping-cough and common catarrh appears by the swelling of the features and the fever. At this time the inflammatory symptoms are stealing upon us, and we have nothing to add to the bold and judicious treatment proposed by Dr. Marcus. This period, in our opinion, lasts a fortnight with more uniformity than the Doctor seems to admit. But there is, probably, a difference from the manner of treatment. We perfectly agree with him that the doctrine of spasm has, in this, as well as many other branches of pathology, been highly destructive to life; and we hail with pleasure the return of Sydenham's and Huxham's practice.

After the inflammatory stage is over, the remedies must be directed by the condition of the patient; and, if the cough becomes chronic, nothing is so much to be depended on as a change of air. But great caution must be used that this air, for the sake of greater purity, is not colder than the former, excepting where the patient has been confined to an artificial atmosphere. In this case, the change must be gradual, and care taken not to expose him to a cold wind. Throughout the whole progress of the disease, we have never found benefit from antimonials or ipecacuanha, or any other such remedies, in doses to induce nausea or vomiting, excepting an occasional emetic, to assist, by pressure on the thorax, in evacuating the bronchiæ; but these attempts should not be oftener repeated than is absolutely necessary.

That the disease is principally seated in the bronchiæ, cannot be doubted: so are many catarrhs, or their sequelæ. But we see no reason, on that account, to alter the same. Hooping-cough may be called a specific disease, having a character different from any other seated in the same parts. Its contagious property, though not to be entirely denied, is certainly different from the laws which govern the exanthemata. On this we shall say more when we have leisure and room to fulfil our promise concerning contagion.

---

### *Edinburgh Medical and Surgical Journal*, No. XLIX. for January, 1817.

### Art. I.—*Critical Review of the State of Medicine during the last Ten Years (continued).*

This article is to be concluded in the succeeding number. We shall therefore defer our remarks till we have the whole before us.

Art.

Art. II.—*On the Causes of the Tropical Endemic, or Yellow-Fever.* By D. J. H. Dickson, M.D. F.L.S. Fellow of the Royal College of Physicians of Edinburgh, &c.

We have before had occasion to remark the diligence and abilities of Dr. Dickson; and the communications with which he has honoured us must be fresh in our reader's memory. We suspect that our correspondence with him has given rise to some parts of the following paper, in which we perceive a progress to greater accuracy in the discrimination of epidemics. We shall endeavour to follow the clue marked out with as much accuracy as possible.

Mr. Sheppard's paper, which we have before had occasion to notice, contains some irrefragable proofs that the yellow-fever may occur a second time in the same subject. Dr. Dickson is ready to admit this. Indeed the possibility of such an event must be admitted, from the author's ætiological inductions.

Marsh miasmata are, by some, considered as the necessary cause of this fever; but, in Dr. Dickson's opinion, this cause has been too much limited. In any situation of the Western tropical regions, the temperature being, for the most part, invariably high, the cause of such a fever may exist from moisture from any cause, on shore, and even on-board, provided it has stagnated long enough, and is exposed to the ardent rays of the sun. Proofs of this are given, quite satisfactory to every inquisitive mind.

"The principal source (continues Dr. D.) of obscurity, and of contradictory opinions on the yellow fever, appears to me to arise from sufficient attention not having been paid to those states of the system upon which the frequency and fatality of the disease so much depend, and which may be designated by the terms susceptibility and disposition. By the first I mean simply the natural liability of the unseasoned European constitution to be affected with that fever in the torrid zone, upon exciting causes sufficiently powerful being applied; by the latter, an acquired or higher degree of susceptibility, by means of which causes less powerful, and otherwise inefficient, become adequate to the production of yellow-fever. The propriety of distinguishing those states, though it may be said they differ only in degree, I hope to make apparent; but I do not mean to say that the terms are the best that could have been selected; it is sufficient, in this case, that they are understood. I have substituted, on reflection, the word disposition for predisposition, (though I should have preferred the more familiar term,) because it might be contended, that the latter ought to imply a previous, rather than an acquired tendency.

"The degree of such disposition will fluctuate considerably during the earlier period of an European's residence in the West

Indies, according to his age, the season of the year, and as various stimuli have a greater or less influence upon the system; or, in other words, in proportion as it has been freely and suddenly, or cautiously and gradually, exposed to their operation. In such a climate, where the youthful sanguine temperament is, at any rate, goaded, by the stimulus of unnatural heat, into a degree of febricular excitement, it is not extraordinary that, from free living, intemperance, or undue exposure or exertion, there should be much danger of this artificial excitation terminating in real fever, until the system becomes gradually inured, and less sensible of such influence by the effect of habit, or assimilated by the supervention of what have been called seasoning, or milder attacks of sickness.

"The dangerous increase of this susceptibility may be often observed in ships recently arrived from Europe, continuing healthy, while refitting in harbour, for ten days, a fortnight, or longer, according to the season, and becoming very sickly afterwards. Its variation and decline are sufficiently exemplified in the disparity of health enjoyed by the crews of ships under repair, at the same time, in the same harbour, and exposed to precisely similar exciting causes, but differing in the length of their residence in a tropical climate, or the degree of exposure to which they had been previously subjected. The variation in these respects will cause such dissimilar results, that a fatal fever will become general, in a short time, in one ship; in another, the sickness will be partial, and less dangerous; while a third will be altogether exempt, or experience only mild and occasional attacks. This gradation will be sufficiently obvious, although its uniformity may be somewhat affected by peculiarities in season, modes of discipline, and various minuter causes, while the chief circumstances are apparently the same.

"The danger of a West-India climate, or, in other words, the tendency to yellow-fever, I conceive, then, to be in the compound ratio of the disposition, and the force of the exciting cause; a weaker exciting cause being sufficient when the system is strongly disposed, and *vice versa*: for, fortunately, these often obtain in an inverse proportion; and the constitution has been more or less habituated, previously to any considerable exposure. How greatly the preservation of health must depend upon the inurement being gradual, is too obvious to require any comment. The degree of security, however, that must be acquired, will be relative; for the insusceptibility will be much greater after an attack of this fever, or from being habituated to miasma, or other remote causes, than from mere length of residence.

"Marshy effluvia, or similar impure emanations in other situations, I have already stated to be, in my opinion, a great source of yellow-fever, either as a predisposing or exciting cause; but, if the above premises be correct, it farther follows, that the causes of yellow-fever may be the same as the remote causes of fever in

 general; general; that they may act in various degrees of intensity, or combination; that the weaker require the aid of disposition to become efficient, but when the system is highly excited, or prepared to fall into fever, that any additional agency, though of itself inoperative and insignificant, may become the occasional cause; and, consequently, that this disease may be called into action, in some cases, by such as are feeble, dissimilar, and so obscure as to elude investigation."

Following a very just maxim, as old as the methodic sect, Dr. Dickson proceeds to shew that, though, in her common operations, according to the Newtonian maxim, Nature delights in simplicity, yet, in disease, more than one cause is necessary. On this occasion we have often heard the maxim of the English philosopher quoted. But it ought to be recollected that Newton was far from conceiving a single cause always sufficient for a physical effect; and his maxim is, that, where we meet with sufficient *causes* to explain the phenomena, we should admit no more.

In pathology, nothing can be added to the remarks of Celsus on the methodic sect of physicians: for, though their opinion of the cause of fever (which we now know to be erroneous) had been well-founded, yet still such a cause solely would prove insufficient. His words are—

"Quod ad Erasistratum pertinet, primum, ipsa evidentia ejus opinioni repugnat; quia raro, nisi post horum aliquid, morbus venit. Deinde non sequitur, ut quod alium non afficit, aut eundem alias, id ne alteri quidem, aut eidem tempore alio noceat. Possunt enim quædam subesse corpori, vel ex infirmitate ejus, vel ex aliquo affectu, quæ vel in alio non sunt, vel in hoc alias non fuerunt, eaque per se non tanta, ut concitent morbum; tamen obnoxium magis aliis injuriis corpus efficiant. Quod si contemplationem rerum naturæ (quam non temere Medici sibi vendicant) satis comprehendisset, etiam illud scisset, nihil omnino ob unam causam fieri, sed id pro causa apprehendi, quod contulisse plurimum videtur. Potest autum id, dum solum est, non movere, quod junctum aliis maxime movet. Accedit ad hæc, quod ne ipse quidem Erasistratus, qui transfuso in arterias sanguine febrem fieri dicit, idque nimis repleto corpore incidere, reperit, cur ex duobus æque repletis, alter in morbum inciderit, alter omni periculo vacarit; quod quotidie fieri apparet. Ex quo disci potest, ut vera sit illa transfusio, tamen illam non per se, cum plenum corpus est, fieri; sed cum horum aliquid accesserit."

This passage shews, in a striking manner, the caution, as well as strength of mind, of that ancient writer, whose object throughout seems to have been, to reduce the science of medicine as nearly as possible to the laws of common sense.

 Dr.

Dr. Dickson produces a number of instances in which fever has been partial, or confined to particular ships, whilst others have been in the same harbour; and that these ships have been mostly fresh arrived, or built recently of green wood, or that the ballast has been ill attended to. After this, some remarks follow on the difference between the contagious property of yellow-fever when compared with plague and typhus. To the latter, he observes, strangers and natives are, *cæteris paribus,* obnoxious in the same degree. This is not strictly the case. The typhus or prison fever does not attack all alike, but, like yellow-fever, those who are accustomed to a different and a better air, are attacked with the greatest certainty and the greatest violence; and it is only when miasma is particularly concentrated, that the inhabitants are affected. Respecting plague, probably Dr. Dickson's opinion may be well founded.

Some judicious remarks follow on the difference between the American insular, and continental climates, and also between them and the Asiatic regions. This leads to the consideration of the fevers which have occurred in the southern parts of Europe, from all which the author considers the inhabitants of the American, and those of southern Europe, in the light of new comers in the West-India islands, because the winter has produced a similar change in their habits as a residence in a colder climate. A well-selected quotation from Humboldt shews how well this was understood by that sagacious philosopher, who very properly remarks, that, when fever occurs at Panama and Callao, it more commonly begins among the crew of vessels from Chili. Not, continues he, that it is brought from thence; but because these people, coming from a peculiarly healthy climate, are more susceptible of any miasma than the natives of the port to which they resort.

"As for the reasons (continues Dr. D.) already given, and from personal observation of the tropical endemic in almost every variety of situation,—proving it to arise in hot, low, moist, close places, whenever new men are exposed to miasmata, intemperance, or fatigue—I must consider the yellow-fever, not as an imported or contagious disease, but as a strictly local and indigenous evil, 'quod sol atque imbres dederant, quod terra crearat sponte sua,' to use the words of Lucretius in a different application. I shall only remark here, that, if it possessed any contagious property, it is to me altogether unaccountable, that conviction thereof should not have been coerced, almost with the force of mathematical demonstration, long before the present day, considering the continual and unrestricted intercourse generally carried on between ships, as well as between the opposite sides of the Isthmus of

Darien.

Darien. But, on the contrary, examples of individual disease, or of a limited number only, are constantly occurring in the same ship, again and again, without extending farther; and it becomes epidemic, as I have endeavoured to explain, only when a generally operating cause produces a general effect. Hence it is legitimately endemic in the West Indies, and becomes often epidemic there at particular seasons, and occasionally, in other countries, after exposure to the influence of tropical heat. If the fever of Gibraltar and other parts of Spain be the same disease, and if it possess any such property, which I consider as still remaining to be proved, I must therefore contend that it is not a native, but an adventitious character, and that, like other diseases attended with febrile action in temperate climates especially, it is susceptible of being modified by the occasional coincidence of peculiar circumstances, such modification placing it in a class which, in my official report on the subject to the Naval Medical Board, (perhaps inaccurately, but for the sake of distinction merely,) I called *diffusible disorders*, the power of dissemination in such not being, as in other communicable diseases, native and inherent, but contingent and acquired. Although I do not mean here to enter farther upon the question of the Peninsular fever, yet, as its progress has been considered by some to be satisfactorily traced, and its prevalence to be unaccounted for by any supposition of an epidemic change of the air, or endemic origin, without a reference to contagion, I may be permitted to remark, in passing, without dwelling upon the inference, that, in the latest work upon the subject, and in which this opinion is temperately supported, the concurrence of a certain height of temperature, and of a combination of circumstances difficult to define, but connected with the climate and individual predisposition,—is nevertheless admitted to be necessary to the existence of the disorder."

We cannot help regretting the obscurity of this passage, in so candid and judicious a writer. To us there appears no reason why causes, as far as we can judge, perfectly similar, should not produce similar effects. If the heat at Cadiz is equal to that in the West-India islands, if Cadiz is a port, and closely built, why should not a similar miasma arise there? The succeeding paragraph seems so entirely introduced in corroboration of such a fact, that we are almost fearful that the author has not courage to speak out.

"Indeed, stronger evidence of a highly deleterious state of the atmosphere, as exemplified by its pernicious influence upon animal life, in these instances at least, cannot well be adduced, than that furnished by the author of the reports himself; for, in the fever at Cadiz in 1800, Sir James Fellowes, I believe in page 45, speaking of the air, says, 'its noxious qualities affected even animals—Canary birds died with blood issuing from their bills;' and he quotes the authority of Arejula in further proof of similar fatal effects effects upon domestic animals, particularly dogs, cats, horses, poultry, and birds."

The next remark is, that a season particularly dry, or particularly wet, is equally free from this dangerous miasma; and that the deleterious principles are evolved during alternate heat and rain, or when heat occurs after rain.

Several observations follow on the supposed indemnity of a constitution which has previously passed through the disease. On this subject we are in want of many facts, before we can admit an established law. Dr. Dickson shews that there exist certain degrees, arising from the intensity of cause, and also from the violence of previous attacks.

Several remarks follow on the mode of treatment, in which the author confirms the modern or rather revived practice of free bleeding and other evacuations; and the whole concludes, with much propriety, in the enforcing of those means by which the fever may be as much as possible prevented.

"During war, when the influx of unseasoned constitutions is considerable, particularly after active service or much exertion, great sickness and mortality are, I fear, unavoidable.

"The healthiness of the navy will very much depend upon avoiding undue exposure to the sun, rain, night air, fatigue, intemperance, and unwholesome shore duties, especially during the sickly season; and upon the attention paid to various regulations, and preventive measures, of which I have had ample opportunities of appretiating and of stating the value, from the inspection, and reports of, generally, between 60 and 70 vessels of war. The bad effects of staying too long in port at one time, and, independent of irregularities, of harbour duties, particularly early in the morning, and after the setting of the sun, as well as during his meridian power, cannot be too strongly adverted to here; and, therefore, a measure of paramount importance is the employment of negroes, natives of the country, or at least of men accustomed to the torrid zone, in wooding, watering, transporting stores, rigging, clearing, careening ships, &c. and, in fine, in all such occupations as must subject them to excessive heat, or noxious exhalations, which cannot fail of being highly dangerous to the health of the unassimilated European. But, when this cannot be done, it will also be greatly preserved by relieving the ships, &c.

"But it will chiefly be preserved by relieving the ships on that station (the prospect of which, alone, has a wonderful effect on the health and spirits of the men) so often, that a foul state of the hold, and the necessity of clearing it in that country, shall, as seldom as possible, arise. During a period of nearly eight years of the late war, considerable sickness and mortality must necessarily have occurred; but, in that time, I have likewise had the great satisfaction of witnessing, in various ships, and on various occasions, that a great degree of health might be maintained in that climate, climate, particularly latterly, when, the season of active warfare being past, the necessity was precluded, and, consequently, the unwholesome duties of clearing the hold, heaving down, or undergoing lengthened repairs in the close harbours of the West Indies, were interdicted; and I am therefore led to conclude, that, to avoid the stronger exciting causes is, to a great extent, to escape the disease."

Such are the outlines of this judicious paper, on which we should have been less diffuse, had not the subject been of late so frequently brought before us. The causes, then, are, intense heat in certain situations, or after rains in all; excessive fatigue; a plethoric condition, or a crowded state of a ship, without sufficient attention to cleanliness and ventilation. The only doubt is, whether these causes, apparently so different, will produce a fever of any marked difference. If we may be allowed an opinion, we should scruple not to say, that, as the same degree of heat is necessary in all, we see no reason why the fever should differ otherwise than in degree. That is, that to all it should be severe according to the degree of heat; to new comers according to the novelty of the stimulus, and to all according to the disposition to plethora and inflammation. The terms Endemic and Epidemic are properly distinguished by the author; but to us it appears, that, whenever we have arrived at sufficient accuracy, the term Endemic will be confined to those diseases which are unconnected with atmospheric peculiarities arising from exhalations. These, we conceive, should be called *epichorii*. But on this we have no room to enlarge at present.—*Habituation* is a subject of very great importance. It is well discussed by our author as far as he goes, but much remains to be learned before any just inductions can be established.

Art. III.—*Case of Pneumonia with Fever of a Bilious Type.* By William Lucas, Surgeon, Stirling.

Art. IV.—*Case of Diseased Arm.* Communicated by Alex. Kennedy, Esq. Madras.

This arm is said, in some respects, to resemble the Barbadoes disease, or Cochin leg. It was, however, different in its abrupt termination; and, after amputation, in the quantity of fluid it contained. In the Barbadoes disease, the substance is found gelatinous, and not fluid. Amputation is also found unsuccessful in the latter complaint.

Art. V.—See our Collectanea.

Art.

Art. VI.—*Remarks on some Remedies which are used in Fevers.* By RICHARD LANPHIER DRAPES, Member of the London College of Surgeons.

These remarks are judicious enough, but not peculiarly striking. However, as far as our own judgment extends, we are glad to find, in the sister kingdom, such a gradual disuse of emetics and diaphoretics; from neither of which, during a long series of practice, have we ever found any advantage, but many real inconveniencies from the use of both, particularly antimonials, when given to any considerable degree. Bleeding and purging are the author's principal remedies.

Art. VII.—*Contributions to Diagnosis.* By MARSHALL HALL, M.D. &c.

These may be as well called cases, excepting the last paper, "on the mode of preparing and preserving vegetable substances," which we shall transcribe.

"It has long been an object of regret, that all the modes of preparing and preserving the vegetable remedies are objectionable, either because the process itself injures the remedy, or because its subsequent preservation is imperfect. Dried vegetable remedies, extracts, tinctures, infusions, and decoctions, are all liable to one or both of these objections. And it is a great desideratum in pharmacy, to discover some mode of preparation that may be free from them.

"The plan which the author would suggest for trial, is that of preparing and preserving these substances, without subjecting them to the previous operation of any external agent whatever, by which their virtues may be impaired. In the case of digitalis, cicuta, hyoscyamus, &c. he has taken the fresh herb, collected free from dew, and, having rubbed the leaves into the finest pulp or paste, he has simply formed a sufficiently consistent mass, by the addition and careful intermixture of dried soap.

"In this manner the vegetable may be long preserved; and the advantage is obtained of administering it throughout the year in its pristine state, and without having previously subjected it to any process, or to the agency of any substance by which its virtues might be destroyed or enfeebled. It may be administered in the form of pills, or mechanically suspended in a draught or mixture.

"Time must be allowed for subsequent experiments on this subject. At present the author contents himself with suggesting this mode of preparing and preserving vegetables to those members of the profession who may feel interested in the inquiry. Perhaps some other article may prove preferable to soap, with the view of preserving vegetable substances without subjecting them to the operation of heat, alcohol, or other chemical agents."

*Des*

*Des Vesanies au Maladies Mentales;* par J. R. Jacquelin Dubuisson, Docteur en Medicine, &c.—*On Mental Diseases;* by J. R. J. Dubuisson, &c. 8vo. Paris, 1816.

Under the sweeping designation of Vesanie (from *Vesania)*, Dr. Dubuisson includes the vast majority of human nature. If the occupation of the mind on an important object produce that want of it in others which we call absence of mind, the person instantly becomes a patient labouring under Vesania. We should, on such grounds, have given a keeper to Sir Isaac Newton, who, having remained in his study two hours after his dinner was served, a friend, waiting for him, and excessively hungry, ate the philosopher's dinner, and covered the dishes again. Sir Isaac at length came in, and, making many apologies, said, "allow me to take my slight dinner, and then I am at your service?" when, lifting the cover, he discovered the skeleton of a chicken; when, turning to his friend, he said, "see what we studious people are: I forgot I had dined."

Though the mania of creating a new nomenclature in France is not less a proof of Vesania than any cited by Dr. Dubuisson, yet, on this occasion, it is praiseworthy. Insanity, so common an expression amongst us, has widely wandered from its primitive and just signification. *Sanitus* (health) was, by extension, applied to mental as well as bodily health; and *insanity*, consequently, signified, from the prefix of the negative preposition *in*, the want of bodily or mental health: but what should we say of a physician, who, finding a person on a bed of sickness, confined by a fever, the gout, &c. declared him *insane?* Though nothing could be more correct than the expression, more just or more simple, and, in itself, devoid of any improper meaning, yet assuredly the medical gentleman, who preferred to such an extent correctness of language, would be cried out against as a foul calumniator and an ignorant practitioner. Instead, therefore, of insanity, which no art will ever bring to its proper acceptation in the opinion of the million, we would recommend the adoption of the word Vesania.

Dr. Dubuisson seems to be of opinion that all men are mad, from the Macedonian to the Swede. If, indeed, the aberrations of reason from the right road to perfection indicate Vesania, it is certainly true that no man alive is in his right senses. Every man who fails in an attempt in which it is possible to succeed, and in which another, with more wit or talent, has succeeded, may, according to Dr. Dubuisson, be fairly pronounced not to be in his right senses. Virtue and vice, it is said, are divided only by a line, line, almost imperceptible. In like manner, good sense and vesania are divided only by a thread: there is no neutral ground. A wise man, or a madman, must be the term; and, what is most painful, it is the *excess* (says Dr. Dubuisson) of learning and wisdom which causes vesania. Not that this is a very new discovery, for we read, in the Acts of the Apostles, that Festus cried out to Paul, "Thou art beside thyself; too much learning doth made thee mad." That vesania, however, may not be held in too much honour, Dr. Dubuisson does not fail to tell us that it is a disorder which poets, painters, politicians, and philosophers, share with tailors, coblers, and weavers. This is unfortunate, for, under the first impression, the sight of a mad-house would inspire all the higher sympathies of the soul: then might we have exclaimed, "There is the unfortunate abode of exuberant genius; there dwell the theorists of the *beau ideal*, of impracticable unattainable perfection!" but to be confounded with tinkers and tailors, wheelwrights and waggoners, destroys all the fine illusions of fancy, and anticipates the functions of the grave, which levels all mankind.

We know not on what data Dr. Dubuisson founds his calculations when he concludes that learned men, politicians, poets, musicians, and painters, with tailors, shoemakers, and weavers, are most liable to attacks of *simple hypochondria*. It is true that, amongst the first class, the continual exercise of the mind upon any subject which requires the exertion of genius or profound meditation, the mind may be so completely absorbed, as may, in the opinion of those who skim all subjects, without fixing the attention upon any one, be supposed to want that proper organization of the faculties which they esteem sound reason; but surely the opinion of those incapable of thinking ought to be received with great limitation, when the question is to decide on the rationality of the studious mind. This would be hurling us back to those dark ages when the people would not suffer their princes to learn to read and write, because these were the acquirements of priests, and tended to enervate the mind. Of this we have a curious instance in the history of the greatest prince of his time, Theodoric, king of the Astragoths, who destroyed the empire of the Cæsars, and seated himself on the throne of Italy, dying without any direct heir, his grandson, the child of his daughter, was destined to succeed him; but the nobles would not suffer him to learn to read and write, because, as they said, it enervated the mind. Too much study may produce baleful effects on the mental system; but who would wish the next generation to be in the state of the barons who signed Magna Charta, who could not not write their names, and were obliged to use a stamp to mark the authenticity of an act (from whence our legal term *sealing* an instrument); or in the situation of that flower of chivalry and companion in arms and glory of our Edward the Third, Sir Walter Maring, who, in order to distinguish the tomb of his father, was obliged to take his chaplain with him to the cemetery, to read the inscription. There might be, and undoubtedly were, fewer instances of mental derangement in these times; but who would purchase the accidental freedom from it on such terms. And, after all, we are yet to learn why a man occupied in stitching a coat, making a pair of shoes, or weaving a piece of cloth, should be liable, more than others, to attacks of *vesania*, or simple hypochondria.

Of *compound hypochondria*, the author enumerates a dozen causes. Unsuccessful love, jealousy, the reverse of fortune, the passion of gaming, disappointed ambition, and religious fanaticism, (says Dr. Dubuisson,) lead to melancholy, with a tendency to suicide; and it is a singular fact that this has sometimes become epidemic. Plutarch tells us of a number of girls, we forget of what place, taking it into their heads to hang themselves; and a few years ago a similar instance of epidemia manifested itself in the town of St. Pierre de Monjau, in the department of the Simplon—a woman hanged herself, and her example was followed by half the women in the parish. Fortunately the Curé put a stop to it, by convincing those who remained that there was neither honour nor pleasure in hanging themselves. The girls of Lyons took a similar whim into their heads, only changing the mode of execution—they drowned themselves.

Partial vesania is when a person reasons well on all subjects but one, like the madman in Don Quixote. In this case the party afflicted fancies himself some other person, as, for instance, a king, a bishop, or an inspired oracle from God. Of the latter class, the world shews, unfortunately, too many examples, in the number of fanatics, which arises from nothing but a derangement of intellectual faculties, naturally weak, operated upon by the superstition and fanaticism of others. Nothing can be so flattering to a mortal as to suppose himself the favourite of the Deity. What he at first wishes, he soon begins to hope, and finishes by believing.

The third class comprises madness with delirium, and madness without delirium. The first may be feared; but the latter most commands our pity. In noticing this third division, we cannot avoid repelling the accusation of madness, charged by French authors on Milton:—that he laboured boured under this class of mental derangement from the vernal to the autumnal equinox; that in the winter he resumed his faculties, but was regularly mad one half the year, until his disorder terminated in blindness.

Incoherence of manner, want of connexion of ideas, confused, vague, and indeterminate notions, without order or connexion—the wildness and extravagance of people so affected, form the symptoms of a disposition to what Dr. Dubuisson distinguishes as the fourth class, viz. *Demence.* This species of mental derangement, observes Dr. Dubuisson, is sometimes allied with great genius. The author cites, on this occasion, the case of the celebrated Guillaume-François Roulle, the father of French chemistry, whose austere probity was only equalled by his profound learning. He was a man of genius, without any other cultivation but in his own particular science; was extremely petulant; his ideas frequently incoherent, and without order and precision; he expressed himself with vehemence, disregarding correctness or neatness in his peroration; he was agitated on his seat, like a person in convulsions, would fall backwards, kick his neighbour's shins, and, without thinking of it, tear the ruffles from his sleeves. One day, in the company of ladies, he started, unbuttoned the knee of his breeches, turned down the stocking, scratched his leg some time, pulled up his stocking, tied his garter, and continued the conversation as if he was perfectly unconscious of what he had been doing. In his lectures, he was generally assisted by his brother and nephew. Sometimes they were absent, and he was obliged to go into the laboratory to find the apparatus necessary. On these occasions he continued the lecture, going and coming; and, if the particular article was not easily found, he had generally finished the lecture on his return. One day, giving his lecture alone, he said, "You see, gentlemen, this vessel on the fire: if I cease to stir it for a single instant, it will explode, and we shall all be blown into the air." At the same instant he ceased to stir it: the explosion took place with a dreadful report. All the windows were broken; but his auditory, of 200, saved themselves in the garden. Fortunately the width of the chimney saved the whole; and the demonstrator only suffered the loss of his wig, the chimney, and the windows of his lecture-room.

The fifth class is Imbecility.—Of the curative means proposed by Dr. Dubuisson we shall give an analysis in a future Number.

*Cursory Remarks on Corpulence; or Obesity considered as a Disease: with a Critical Examination of Ancient and Modern Opinions, relative to its Causes and Cure. Third Edition, containing a Reference to the most remarkable Cases that have occurred in this Country.* By WILLIAM WADD, Surgeon. 8vo. pp. 129. Callow.

THE abilities of Mr. Wadd are so well known, that we need scarcely remind our readers of his valuable treatise on Strictures, in which he has revived the Hunterian doctrine and practice, after they had been for a time nearly superseded by the caustics of Sir E. Home and his followers. Nor need we mention Mr. W.'s representations of Diseased Bladder, Testicles, and their Appendages, illustrated by his own etchings. It gives us particular pleasure when such an author undertakes what is often considered a lighter subject. Nothing, it is true, in physiology, can be light, if it leads to pathology. Of this the venerable Heberben gave a striking conviction, when the same person, in the same compilation, favoured the world with the first description of Angina Pectoris, and the first scientific account of Chicken-pox.

As is usually the case, an accidental circumstance led the author into these inquiries.

"The Remarks on Corpulency (says he, in a Preface) first appeared with a confession that they had never been prepared for the public eye. For that reason they were published without a name.

"In this imperfect state they passed through two impressions; and, as no pains were taken to conceal the author, he soon became generally known. It was, therefore, his wish to render the work more systematic; but professional duties, and publications, have prevented his attempting more than to arrange such facts as have occurred in his practice or reading. They have gradually accumulated; and, judging of the importance of the subject, by the reception with which such a trifle has been honoured, he is induced to submit them again to the corpulent good-humoured part of the community, in their present shape."

The work commences with some general remarks on the anatomy and physiology of those parts in which fat is secreted—the manner in which it is distributed—the various opinions of the most celebrated anatomists, foreign and British, on these subjects—the characters in whom fat chiefly accumulates—the probable causes of such accumulations. All this leads to consideration of the means by which comeliness may be acquired and corpulency avoided. We extract the following, which, though among the lighter parts of these remarks, is more unconnected, and, consequently,

 more more readily comprehended, without a previous knowledge of the question.

"Nor will any of the other medicaments proposed afford better prospects of success. As auxiliaries they may occasionally be useful; but the only certain and permanent relief is to be sought in a rigid abstemiousness, and a strict and constant attention to diet and exercise. 'J'ai pour garants de mon sentiment, les medicins les plus fameux tant anciens, que modernes.'

"The ancients were by no means inattentive to these instruments of medicine. Herodicus is said to have been the first who applied the exercises and regimen of the gymnasium to the removal of disease, or the maintenance of health. Celsus refers us to Asclepiades, as the physician who introduced friction into the Roman practice; remarking, at the same time, that he did no more than revive, with some improvements, the precepts of Hippocrates, who has said, that, by friction, if violent, the body may be rendered harder—if gentle, softer—if frequent, that it may be lessened or extenuated—if moderate, that it may be filled or rendered more sleek. Hence, it follows, continues Celsus, that this remedy, if used with judgment, may be applied with advantage in any condition of the body: it certainly is a remedy too much overlooked by the moderns in colder climates. Mr. Grosvenor has turned it to much advantage in many stubborn local complaints.

"Every cure was commenced by a rigid enforcement of the *diatrition*, or three days entire abstinence, which was resumed, in obstinate cases, a second and a third time, after intervals barely sufficient to allow as much nourishment as might keep the patient from dying of hunger and thirst.*

"This old-fashioned practice would be an excellent mode of treating some of our new diseases; and, in truth, some modern philosophers seem to think so. Abstinence from animal food was considered a moral duty, by the learned Ritson, ten years ago; and we have very lately had an erudite exhortation to 'return to nature,' and vegetable diet, by a gentleman whose whole family live according to the following bill of fare. 'Our breakfast,' he observes, 'is composed of dried fruits, whether raisins, figs, or plums, with toasted bread, or biscuits, and weak tea, always made of distilled water, with a moderate portion of milk in it. The

"* We might almost suppose our early legislators had taken a hint from the ancient doctors. By a statute of Edward I. felons sent to suffer 'Prison forte et dure,' were committed 'ad dietam'—a term ironically expressive of the said sustenance the sufferer was allowed; viz. on the first day, three morsels of the worst bread; on the second, three draughts of water from the next puddle: this was alternately his diet till he died. Gloomy as this retrospect is, it might have proved an important document, if any register could inform us how long life could be protracted by such means."

water children, who do not seem to like the flavour of tea, use milk and water instead of it. When butter is added to the toast, it is in very small quantity. The dinner consists of potatoes, with some other vegetables, according as they happen to be in season; macaroni, a tart, or a pudding, with as few eggs as possible: to this is sometimes added a dessert. Onions, especially those from Portugal, may be stewed with a little walnut pickle, and some other vegetable ingredients, for which no cook will be at a loss, so as to constitute an excellent sauce for all other vegetables. As to drinking, we are scarcely inclined, on this cooling regimen, to drink at all; but when it so happens, we take distilled water, having a still expressly for this purpose in our back kitchen.'—*Newton's Return to Nature*, p. 144.

"The article of *drink* requires the utmost attention. Corpulent persons generally indulge to excess; if this be allowed, every endeavour to reduce them will be vain.

"Newmarket affords abundant proofs how much may be done by exercise. Jockies sometimes reduce themselves a stone and a half in weight in a week; and we learn, by the answer of a person well versed in the business of training to the question 'Would he recommend a similar process to reduce corpulency in other people, whether male or female?' 'That he would recommend a similar process to reduce corpulency in either sex, *as, from experience, he perceives, that the constitution does not appear to be injured by it.*'*

"Herodicus, in ancient times, ordered a patient to walk from Athens to Megara, a distance of twenty miles, with a strong injunction to walk back again as soon as he had touched the walls.

"Many would willingly submit to any violent remedy, so that an immediate benefit could be produced; but, unless the disease speedily gives way, they despair of success, consider it as unalterably connected with their constitution, and, of course, return to their former habits. This feeling is too often encouraged by the ill-judged advice of friends, who thus become unthinking accomplices in the destruction of those whom they esteem and regard."

The reader will see, by this, and by Mr. Wadd's ingenious remarks on Sir E. Home's paper on the Formation of Fat, that the work retains some of the same original pleasantry, as when written more for common than for professional readers. The more scientific remarks, however, are so connected with the whole, that we find it difficult to extract them; and must conclude with recommending the perusal of the work to such of the profession as feel themselves at a loss, when urged by their patients to relieve them from their superfluous *sarcoma*.

"* Vide Code of Health, by Sir John Sinclair."

The

*The Botanist's Companion, or an Introduction to the Knowledge of Practical Botany, and the Uses of Plants, either growing Wild in Great Britain, or cultivated for the Purposes of Agriculture, Medicine, Rural Economy, or the Arts.* By WILLIAM SALISBURY, of the Botanic Garden, Sloane-street. 2 vols. small 8vo.—Longman and Co.

THE prospect of an early spring has induced us to take notice of this useful little compendium sooner than we intended. The advantages of botany are much greater than young men are aware of. The bare knowledge of a few officinals, and of the means of distinguishing poisonous from salutiferous plants is very properly all that is required of the examiners at Apothecaries' Hall. Indeed, the extent of the medical art, and the expenses attending it, are sufficient to alarm any one who looks to the mere profits; and, it must be admitted, that a scientific knowledge of botany is by no means an essential. But, in the choice of every profession, three things are to be considered:—First, a conscientious discharge of those duties, and an attention to all those means by which we may be qualified for the arduous employment we have in view; next, the means which may facilitate our professional introduction into the world; and, lastly, those auxiliaries which, though slightly connected with our profession, are not inconsistent with it, and may serve to render our labours more supportable, and oftentimes agreeable. For the two last purposes, nothing furnishes so ample and ready a scope as the study of botany. The public can rarely and very slowly judge of a man's medical knowledge; but all the ladies, whose good wishes are the most to be desired, will feel interested by his botanical instructions. Chemistry is now become a science, which, if followed to any extent, must occupy a man's whole thoughts, and detach his mind from more necessary pursuits. Even a residence in the metropolis, or some large town, is necessary, as well as a perpetual intercourse with others engaged in the same inquiries. Botany, on the contrary, is the amusement of the village practitioner, and relieves his journeys from house to house by the prospect of every hedge as he passes it. It is also not progressive, like those sciences which depend on experiment, and may, therefore, be retained, and even improved, without a perpetual recurrence to the labours or improvement of others. In a word, it is an elegant amusement, partaking of that science to which a medical man devotes himself.

But, however simple it may be, its fundamental principles must be learned, and its practical rules acquired, in order to to render it interesting, or to enable us to improve ourselves or others. Like every other study, its institutes should be acquired perfectly, but this only may be reduced to practice at the same time. This is not to be accomplished during the hurry of a winter's campaign, at the hospitals, dissecting rooms, and medical theatres. A summer's residence in town cannot, therefore, be better occupied than by a careful selection of the winter occurrences, and the application to botany. By these means, the student will return fresh to his labours as the autumn advances, and carry with him, into the country, an accomplishment which will render his society more desireable, add to his professional reputation, and very much increase the sources of his own enjoyments. At the same time, we would advise every young practitioner to be very careful how he brings forward his botanical knowledge too early. He may acquire the character of a naturalist, instead of a physician or surgeon. Let him, therefore, be careful to undervalue such an acquisition, even if he finds it useful or necessary to bring it forward, and let him speak of it as it really should be, rather the amusement of his leisure than the employment of his graver hours.

Having said thus much in favour of botany as a study, or, if our readers please, as an accomplishment, we need add but little more of the work before us, than that it is admirably calculated to keep up or revive all the knowledge a student may have acquired during his stay in London. Our readers are well acquainted with Mr. Salisbury's botanical excursion, than which we know not a more rational means of passing a summer's day, and we cannot explain the author's intention better than in his own words.

"As the practice of horticulture and agriculture on an extensive scale has been the employment of my life, in which I have had occasion to investigate minutely the useful and noxious qualities of plants of every description, as well as their distinguishing characters; and having, of course, had more opportunity of acquiring this knowledge than usually falls to the lot of most men; I trust it will not be presumed altogether improper in me to attempt to instruct persons by a comparatively easy method in attaining a knowledge of the science of botany. And, without meaning in the least to derogate from the merits of the learned and highly respected professors, who, of late years, have taught this science, I beg leave to observe, that the lectures usually delivered for this purpose, have, in a great measure, failed of the intended object. For, although the first principles may be in that manner explained, yet a perfect and useful acquaintance with this delightful part of natural history can be acquired only by reading in the book of nature, by paying proper attention to the different plants in their native habitats, and by having their true characters demonstrated, in the regular progress of their growth to maturity.

"Having taken upon me this task, in a method peculiar to myself, I found it necessary to have a small work which might contain the principles of classification according to Linnæus, the terminology, and, at the same time, a concise arrangement of all the plants according to those principles, with their discriminating characters, and the various uses to which each is applicable. The want of such a work has been so often expressed by those who have honoured me with their attendance in my botanical excursions, that it will require but little apology on my part, for intruding this small work on the public. Should it chance to fall under the notice of those whose business it is to investigate critically the merits of such productions, I must intreat of them to reflect, that it was written for the purpose of instructing persons who are ignorant of, and desirous of obtaining, a knowledge of the subjects it treats on; and no one, perhaps, who has not taken upon himself the task of learning this science, or of teaching others, will, at first, be capable of estimating its utility.

"It is, in fact, only by considering what are the wants of a student, that its fitness for the purpose of teaching botany can be estimated; and those who have attempted to study the science with our present helps will, I trust, acknowledge the utility of this work in forwarding their pursuit. In the several arrangements, it will be observed, that I have departed from the modes usually adopted; and this has been done chiefly for the purpose of rendering it the more concise, it being intended chiefly as a pocket compendium."

The first volume comprehends,

"A concise Introduction to the Study of Botany—On the Classes—On the Orders—On the Genera; or, the Parts of Fructification, with a particular Description of each—The Application of the foregoing for the Discovery of the Genera—The Species of Plants, and on the Characters which generally constitute their Distinctions—Botanic Terms as relative to the Root and Trunk; Branches, Leaves, Fulcra, Inflorescence, Fructification, Bud, Bulb, Vernation, Measures—A General Arrangement of the British Genera, under their respective Classes and Orders—Table of Abbreviations, and an Alphabetical Arrangement of Species of British Plants, with their descriptive Characters, Place of Growth, Season of Flowering, and Reference to their Useful and Noxious Properties, as described in the Second Volume of this Work."

The second volume contains plants useful in agriculture, comprehending the grasses and grains; to which are added, those articles principally used in dying and cloathing, and a few others. A chapter follows, in which the last subject is taken up more comprehensively; including medicinal plants, whether contained in any of the Pharmacopœias or not, and also the mode of drying and preserving them; plants for culinary culinary purposes, whether wild or cultivated, &c. Next poisonous plants, distinguishing their properties, as

"Bitter Nauseous Poisonous Plants—Acrid Poisonous Vegetables—Stupefying Poisonous Vegetables—Fœtid Poisons—Drastic Poisons—Poisonous Fungi, Mushrooms, &c."

A very useful addition to this chapter contains an enumeration of certain plants which are found noxious to cattle, but which their instinct does not teach them to refuse. An appendix concludes the work, containing every necessary article which could not be readily comprehended under any other division.

Such is this useful little compilation, which may, with much propriety, be called the Country Practitioner's Vade Mecum; for, if there is a study of which he may say with the philosopher, *equitantur nobiscum!* it is botany.

---

### MEDICAL AND PHILOSOPHICAL INTELLIGENCE.

*To the Editors of the London Medical and Physical Journal.*

GENTLEMEN,

